# Risk factors for injuries in New Zealand older adults with complex needs: a national population retrospective study

**DOI:** 10.1186/s12877-021-02576-1

**Published:** 2021-11-04

**Authors:** Rebecca Abey-Nesbit, Philip J. Schluter, Tim J. Wilkinson, John Hugh Thwaites, Sarah D. Berry, Heather Allore, Hamish A. Jamieson

**Affiliations:** 1 Christchurch School of Medicine and Health Sciences, University of Otago, Christchurch, New Zealand; 2 School of Health Sciences, University of Canterbury–Te Whare Wānanga o Waitaha, Christchurch, New Zealand; 3 School of Clinical Medicine – Primary Care Clinical Unit, The University of Queensland, Brisbane, Australia; 4 Canterbury District Health Board, Christchurch, New Zealand; 5 Harvard Medical School, Boston, MA USA; 6 Division of Geriatric Medicine, Department of Medicine, Beth Israel Deaconess Medical Center, Boston, MA USA; 7 Hebrew Senior Life, Boston, MA USA; 8 Department of Biostatistics, Yale School of Public Health, New Haven, CT USA; 9 Department of Internal Medicine, School of Medicine, Yale University, New Haven, CT USA

**Keywords:** Falls-related injuries, Geriatric assessment, Older adults, interRAI, Home care

## Abstract

**Background:**

Falls and falls-related injuries are common among older adults. Injuries in older adults lead to poor outcomes and lower quality of life. The objective of our study was to identify factors associated with fall-related injuries among home care clients in New Zealand.

**Methods:**

The study cohort consisted of 75,484 community-dwelling people aged 65 years or older who underwent an interRAI home care assessment between June 2012 and June 2018 in New Zealand. The injuries included for analysis were fracture of the distal radius, hip fracture, pelvic fracture, proximal humerus fracture, subarachnoid haemorrhage, traumatic subdural haematoma, and vertebral fracture. Unadjusted and adjusted competing risk regression models were used to identify factors associated with fall-related injuries.

**Results:**

A total of 7414 (9.8%) people sustained a falls-related injury over the 6-year period, and most injuries sustained were hip fractures (4735 63.9%). The rate of injurious falls was 47 per 1000 person-years. The factors associated with injury were female sex, older age, living alone, Parkinson’s disease, stroke/CVA, falls, unsteady gait, tobacco use, and being underweight. Cancer, dyspnoea, high BMI, and a decrease in the amount of food or fluid usually consumed, were associated with a reduced risk of sustaining an injury. After censoring hip fractures the risks associated with other types of injury were sex, age, previous falls, dyspnoea, tobacco use, and BMI.

**Conclusions:**

While it is important to reduce the risk of falls, it is especially important to reduce the risk of falls-related injuries. Knowledge of risk factors associated with these types of injuries can help to develop focused intervention programmes and development of a predictive model to identify those who would benefit from intervention programmes.

## Background

Fall-related injuries sustained by older adults have worse outcomes than in younger individuals [1, 2]. Due to complications of ageing, older adults who sustain an injury are more likely to suffer from decreased function, disability, chronic pain, long stays in hospital, and increased mortality [1–3]. Falls and fall-related injuries have been noted to be increasing after adjusting for the increasing age of the global population, and this increase in falls is likely associated with an increase in fall-related injuries such as hip fractures [4–6]. Due to the seriousness of these injuries, the New Zealand government has made it a priority to improve health outcomes associated with injurious falls [7].

Standardised assessments have been mandated in New Zealand since 2012 to identify home health care needs of community-dwelling older adults. New Zealand uses interRAI-home care (HC) assessments to identify what areas of health care an individual requires. The interRAI-HC assessment contains 236 questions across 20 domains, including cognition, psychosocial factors, health conditions, self-reported falls history, and skin conditions. A copy of the interRAI-HC form can be obtained from the interRAI Home Care Assessment Form and User’s Manual [8]. All New Zealanders who require publicly funded home care services must undergo an interRAI-HC assessment [9].

Fall-related injuries in older adults are a global concern and have been studied widely within the literature. Risk factors identified from previous research include recent hospitalisations, previous history of falls, stroke, Parkinson’s disease, lower limb amputations, cognitive impairment, arthritis, and diabetes [10–18]. Additionally, there have been other studies in New Zealand examining hip fracture incidence rates [19–22]. However, hip fractures are not the only injuries from falls. Few studies have identified risk factors for other significant fall-related injuries such as pelvic fractures, fractures of the distal radius, or proximal humerus fractures. A 2017 study found that falling onto low-impact flooring reduced the number of fall-related injuries compared with falling onto standard vinyl flooring [23]. Elley et al. conducted a trial on community-based older adults who had a previous fall, and they found there was no difference in the number of falls an individual had, after nurse-led interventions were put in place [24]. However, an earlier study conducted by Campbell et al. found that falls and fall-related injuries were reduced in women aged 80 years and older who took part in a tailored exercise programme [25].

Work on reducing falls has been conducted, but it is also important to work towards reducing fall-related injuries [26]. One way to achieve this is by identifying risk factors associated with fall-related injuries and to see if they are different from those that predict hip fractures. The primary objective of our study was to determine risk factors for trauma related injuries in community-dwelling older people receiving home care services in New Zealand. Our secondary objective was to identify risk factors for the non-hip fracture injuries.

## Methods

### Study design

We conducted a retrospective time-to-event study from a national cohort.

### Participants

Participants included New Zealand resident adults aged 65 years and older who had an interRAI-HC assessment between 1 July 2012 and 1 June 2018. All participants were living in the community at the time of their assessment and consented for their information to be used for planning and research purposes. Where an individual had multiple assessments, the first interRAI-HC assessment was utilised. We randomly selected 75,484 individuals for the development cohort.

### Instruments/variables

The interRAI-HC version 9.1 (© interRAI corporation, Washington, D. C., 1994–2009) assessment tool is a comprehensive geriatric assessment that consists of 236 questions across 20 domains including Mood and Behaviour, Functional Status, Disease Diagnoses, and Oral and Nutritional Status [27, 28]. In New Zealand it has been mandated that this assessment tool be used for all older adults who require publicly funded home care services or for people entering aged residential care. Health practitioners refer individuals for an interRAI-HC to assess their health care needs. Each assessment is carried out by a trained interRAI assessor who has undergone rigorous training. The assessment will usually be carried out in the individual’s home and the assessor uses a variety of sources to complete assessments; including, checking the patient’s medical records, interviewing the individual and sometimes their family members [29]. The assessment data are entered into an electronic database and the data are stored by New Zealand’s Technical Advisory Services (TAS).

Approximately 93% of people undergoing an interRAI-HC assessment consent for their information to be used for planning and research purposes [27]. The assessments can be linked to other health datasets such as mortality and hospital admissions using a National Health Index (NHI) number. An NHI number is a unique identifier allocated to anyone who receives health care services in New Zealand [30].

Within the interRAI-HC assessment, individuals may choose up to three ethnicities; for analysis purposes we employed priority coding according to New Zealand’s Ministry of Health (MoH) guidelines [31]. Where an individual indicated more than one ethnicity priority was given to Māori, then Pasifika, and then Asian ethnicities.

Hospital admissions data for the injuries of interest were obtained from the National Minimum Dataset (NMDS) [32], released from the MoH with encrypted NHI numbers for all consenting interRAI-HC participants. Injuries chosen for this study were identified as common fall-related injuries within the literature and only those injuries that led to the individual being admitted to hospital. For instance, people who sustain a wrist fracture are less likely to be admitted to hospital. The injuries included for analysis were fracture of the distal radius, hip fracture, pelvic fracture, proximal humerus fracture, subarachnoid haemorrhage, traumatic subdural haematoma, and vertebral fracture. Injuries were identified using ICD-10-AM (International Statistical Classification of Diseases and Related Health Problems, Tenth Revision, Australian Modification) diagnostic codes I60 I62.0 S22 S32 S52.2 S42.3 S49.0 S720 S721 S722 S723 S724 S728 and S729. Ecodes (External cause of injury codes) were unavailable for all hospital admissions and were unable to be utilised. The first hospital admission after an individual’s assessment was used. The hospital admission dataset contains up to 20 different diagnosis codes. For cases where the individual sustained multiple injuries of interest, the first instance of one of the injuries listed above was noted down. When censoring individuals with hip fracture if there was any instance of hip fracture in any of the 20 diagnostic codes, the individual was censored from analysis. Mortality data were provided by the MoH from their Mortality Collection (MORT) [33] and included encrypted NHIs so they could be matched to the interRAI-HC assessments.

### Statistical analysis

Reporting of the results adhered to RECORD (REporting of Studies Conducted using Observational Routinely collected Data) guidelines [34] to ensure this study reports results accurately and clearly. Basic frequency distributions of each variable of interest were reported. Multicollinearity was tested for by examining the variance inflation factor of each variable of interest. Most questions within the interRAI-HC are mandatory and therefore there was very little missing data. Body Mass Index (BMI) had a high percentage of missing values and these were incorporated into an ‘Unknown’ category. All other missing variables were included in analysis due to their low numbers. Risk factors for injuries were determined using competing risk regression models with the Fine and Grey method [35], injurious falls were the failure event and death was the competing event. Unadjusted models of each variable of interest were utilised and an adjusted model including all variables and adjusting for age, sex, and ethnicity were conducted. Subhazard ratios (SHRs) and 95% confidence intervals (CIs) were reported for each variable in the model. A competing risk regression model treating hip fracture as a censored event was also conducted to identify whether there was a difference between the risk factors for hip fracture and other injuries of interest. IBM SPSS version 26 [36] was used for descriptive analyses and data cleaning, and Stata IC version 16 [37] was used to run competing risk regressions, and α = 0.05 defined statistical significance.

### Ethics

Ethics permission for this study was granted by the Ministry of Health’s Health and Disability Ethics Committees (14/STH/140). Only those individuals who consented to their data to be used for research purposes were included in this study.

## Results

### Participants

**Fig. 1:**
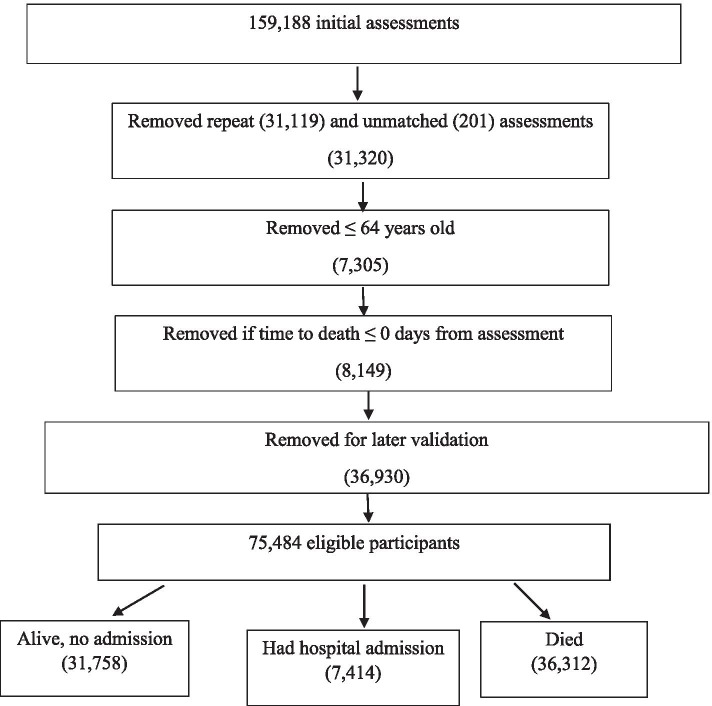
Exclusion criteria for interRAI HC assessments

After exclusions were applied, there were a total of 75,484 participants. Figure 1 below, details the exclusion criteria.

### Demographics

Participant’s mean age was 82.1 years (range: 65 to 107 years), with 44,939 (59.5%) females. Approximately 87.5% (66080) of people identified as European ethnicity and 5.8% (4348) people identified as Māori. Approximately 61.9% (46733) of people required an assistive device such as a cane, walker, or pushing wheelchair. Over half of individuals reported minimal to moderate levels of fatigue (58.2%).

### Injuries and deaths

Within the cohort over the 6-year period 7414 (9.8%) sustained an injury, 36,212 (48.0%) died and the remaining 31,578 (41.8%) had not been admitted to hospital for a fall-related injury or died by the end of the study period. Most injuries sustained were hip fractures (4735 63.9%), followed by fractures of the proximal humerus (735 9.9%). A full list of injuries and their frequencies can be found in Table 1. Median follow-up time after the interRAI-HC assessment was 21.5 months (25th percentile 8.5 months, 75th percentile 39.0 months) from assessment, with a total person-time of 157,134 years and the rate of injurious falls was 47 per 1000 person-years.

**Table 1:** Distribution of injury type sustained

Injury Type	Number (%)
Distal radius fracture	640 (8.6)
Hip fracture	4735 (63.9)
Pelvic fracture	470 (6.3)
Proximal humerus fracture	735 (9.9)
Subarachnoid haemorrhage	160 (2.2)
Subdural haematoma	303 (4.1)
Vertebral fracture	371 (5.0)

### Unadjusted and adjusted analyses

The results of the unadjusted and adjusted analyses for the demographic variables are displayed in Table 2. People who were living with others were less likely to sustain an injury than those who lived alone (SHR 0.94 95% CI: 0.89–0.99). The risk of sustaining a fall-related injury increased for those aged 65 to 94 years, but those who were aged 95 years and older were slightly less likely to sustain an injury than those aged between 85 and 94 years. Females were more likely to sustain an injury than males (SHR: 1.31 95% CI: 1.24–1.38).

**Table 2:** Demographic frequencies for each event and unadjusted and adjusted competing risk regression results

		First Event				
Variable names	Total n (%)	Alive, no admission	Hospital admission	Death	Unadjusted	Adjusted^b^
Sex^a^						
Male	30,497 (40.4)	11,155 (36.5)	2387 (7.8)	16,955 (55.7)	1 (Reference)	1 (Reference)
Female	44,939 (59.6)	20,575 (45.8)	5026 (11.2)	19,338 (43.0)	1.45 (1.38 1.53)	1.31 (1.24 1.38)
Age Group (years)						
65–74	13,485 (17.9)	7349 (54.5)	828 (6.1)	5308 (39.4)	1 (Reference)	1 (Reference)
75–84	34,862 (46.2)	15,669 (44.9)	3211 (9.2)	15,982 (45.8)	1.48 (1.37 1.59)	1.32 (1.22 1.44)
85–94	25,497 (33.8)	8391 (32.9)	3155 (12.4)	13,951 (53.8)	1.98 (1.84 2.14)	1.49 (1.37 1.63)
95+	1640 (2.2)	349 (21.3)	220 (13.4)	1071 (65.3)	2.10 (1.82 2.44)	1.39 (1.19 1.62)
Ethnicity						
Māori	4348 (5.8)	2023 (46.5)	234 (5.4)	2091 (48.1)	1 (Reference)	1 (Reference)
Pacific People	2388 (3.2)	1242 (52.0)	125 (5.2)	1021 (42.8)	0.94 (0.75 1.16)	0.93 (0.74 1.17)
Asian	1954 (2.6)	1037 (53.1)	159 (8.1)	758 (38.8)	1.58 (1.29 1.92)	1.19 (0.97 1.47)
European	66,080 (87.5)	27,061 (41.0)	6827 (10.3)	32,192 (48.7)	1.90 (1.67 2.16)	1.54 (1.35 1.77)
Other	714 (0.9)	395 (55.3)	69 (9.7)	250 (35.0)	1.88 (1.43 2.45)	1.68 (1.28 2.21)
Living Arrangement						
Lives alone	36,599 (48.5)	15,612 (42.7)	4021 (11.0)	16,966 (46.4)	1 (Reference)	1 (Reference)
Lives with others	38,885 (51.5)	16,146 (41.5)	3393 (8.7)	19,346 (49.8)	0.80 (0.76 0.83)	0.94 (0.89 0.99)

^a^48 values missing ^b^Variables adjusted for age, sex, and ethnicity

Table 3 presents frequency distributions and the unadjusted and adjusted SHRs for each variable of interest. Risk factors for injuries were sex, age, living alone, Parkinson’s disease, stroke/CVA, falls, unsteady gait, tobacco use, and being underweight. Cancer, dyspnoea, high BMI, and a decrease in the amount of food or fluid usually consumed, were associated with a reduced risk of sustaining an injury.

**Table 3:** Variables of interest frequencies for each event and unadjusted and adjusted competing risk regression results

		First Event				
Variable names	Total n (%)	Alive, no admission	Hospital admission	Death	Crude SHR (95% CI)	Adjusted*
Cognitive Skills^b^						
Independent	35,030 (46.4)	15,702 (44.8)	3305 (9.4)	16,023 (45.7)	1 (Reference)	1 (Reference)
Minimal Independence	28,645 (37.9)	12,089 (42.2)	2837 (10.0)	13,719 (47.9)	1.10 (1.05 1.16)	1.01 (0.96 1.07)
Moderate to Severe dependence	11,772 (15.6)	3964 (33.7)	1269 (10.8)	6539 (55.5)	1.16 (1.08 1.23)	1.00 (0.96 1.07)
No discernible consciousness	36 (0.0)	2 (5.6)	3 (8.3)	31 (86.1)	0.82 (0.26 2.56)	1.39 (0.18 11.0)
Hearing loss^d^						
Adequate	39,339 (52.1)	18,174 (46.2)	3625 (9.2)	17,540 (44.6)	1 (Reference)	1 (Reference)
Minimal to moderate	33,257 (44.1)	12,752 (38.3)	3466 (10.4)	17,039 (51.2)	1.048 (0.99 1.11)	1.03 (0.98 1.09)
Severe to none	2870 (3.8)	831 (29.0)	322 (11.2)	1717 (59.8)	1.04 (0.99 1.10)	1.00 (0.89 1.13)
Vision loss^e^						
Adequate	54,518 (72.2)	24,231 (44.4)	5128 (9.4)	25,159 (46.1)	1 (Reference)	1 (Reference)
Minimal to moderate	19,267 (25.5)	6973 (36.2)	2094 (10.9)	10,200 (52.9)	1.12 (1.07 1.17)	1.00 (0.94 1.05)
Severe to none	1680 (2.2)	553 (32.9)	190 (11.3)	937 (55.8)	1.16 (1.03 1.29)	0.98 (0.85 1.13)
Walking^g^						
Independent	57,273 (79.0)	27,226 (47.5)	5136 (9.0)	24,911 (43.5)	1 (Reference)	1 (Reference)
Some assistance required	11,175 (15.4)	3041 (27.2)	1466 (13.1)	6668 (59.7)	1.44 (1.36 1.52)	1.03 (0.90 1.19)
Maximum Assistance/Dependent	4028 (5.6)	790 (19.6)	532 (13.2)	2706 (67.2)	1.43 (1.31 1.56)	1.22 (0.99 1.49)
Locomotion^h^						
Independent	57,883 (78.3)	27,446 (47.4)	5202 (9.0)	25,235 (43.6)	1 (Reference)	1 (Reference)
Some assistance required	10,667 (14.4)	2970 (27.8)	1409 (13.2)	6288 (58.9)	1.46 (1.38 1.55)	1.04 (0.91 1.20)
Dependent	5338 (7.1)	1062 (19.9)	642 (12.0)	3634 (68.1)	1.30 (1.20 1.40)	0.84 (0.69 1.03)
Primary Mode of Locomotion^i^						
Walking, no assistive device	24,712 (32.7)	13,202 (53.4)	1643 (6.6)	9867 (4.0)	1 (Reference)	1 (Reference)
Assisted walking	46,733 (61.9)	17,622 (37.7)	5397 (11.5)	23,714 (50.7)	1.69 (1.60 1.79)	1.32 (1.24 1.41)
Unable to walk	4037 (5.3)	932 (23.1)	374 (9.3)	2731 (67.6)	1.31 (1.17 1.46)	1.18 (0.96 1.47)
Timed 4 Metre walk^i^						
0–15 s	47,062 (62.3)	23,007 (48.9)	4168 (8.9)	19,887 (42.3)	1 (Reference)	1 (Reference)
16–29 s	5662 (7.5)	2093 (37.0)	689 (12.2)	2880 (50.9)	1.24 (1.48 1.35)	1.08 (1.00 1.18)
30+ seconds	5252 (7.0)	1858 (35.4)	666 (12.7)	2728 (51.9)	1.23 (1.13 1.33)	1.09 (1.00 1.18)
Incomplete test	17,506 (23.2)	4798 (27.4)	1891 (10.8)	10,817 (61.8)	1.15 (1.09 1.21)	0.99 (0.92 1.06)
Total hours of exercise or physical activity^i^						
None/Less than 1 h	42,971 (56.9)	16,408 (38.2)	4019 (9.4)	22,544 (52.5)	1 (Reference)	1 (Reference)
1–4 h	29,476 (39.1)	13,861 (47.0)	3094 (10.5)	12,521 (42.5)	1.09 (1.04 1.14)	1.06 (1.01 1.11)
4 h or more	3035 (4.0)	1487 (49.0)	301 (9.9)	1247 (41.1)	0.90 (0.80 1.02)	1.04 (0.92 1.17)
Number of days left house in last 3 days^i^						
None	25,432 (33.7)	7309 (28.7)	2939 (11.6)	15,184 (59.7)	1 (Reference)	1 (Reference)
1–2 days	20,473 (27.1)	9027 (44.1)	1968 (9.6)	9478 (46.3)	0.85 (0.81 0.90)	1.04 (0.97 1.11)
3 days	29,577 (39.2)	15,420 (52.1)	2507 (8.5)	11,650 (39.4)	0.75 (0.71 0.79)	1.02 (0.95 1.09)
Bladder Continence^j^						
Continent	47,560 (63.1)	21,212 (44.6)	4423 (9.3)	21,925 (46.1)	1 (Reference)	1 (Reference)
Infrequently incontinent	7219 (9.6)	3116 (43.2)	740 (10.3)	3363 (46.6)	1.11 (1.03 1.20)	0.98 (0.91 1.07)
Occasionally incontinent	6724 (8.9)	2697 (40.1)	724 (10.8)	3303 (49.1)	1.16 (1.07 1.25)	0.97 (0.90 1.05)
Frequently Incontinent	11,267 (14.9)	4108 (36.5)	1223 (10.9)	5936 (52.7)	1.15 (1.08 1.22)	0.97 (0.91 1.04)
Incontinent	2653 (3.5)	614 (23.1)	299 (11.3)	1740 (65.6)	1.14 (1.01 1.28)	1.04 (0.89 1.21)
Bowel Continence^k^						
Continent	63,166 (83.9)	28,185 (44.6)	6086 (9.6)	28,895 (45.7)	1 (Reference)	1 (Reference)
Infrequently incontinent	4936 (6.6)	1741 (35.3)	490 (9.9)	2705 (54.8)	1.02 (0.93 1.12)	0.97 (0.88 1.06)
Occasionally Incontinent	3829 (5.1)	1056 (27.6)	438 (11.4)	2335 (61.0)	1.15 (1.05 1.27)	1.02 (0.91 1.13)
Frequently Incontinent	1852 (2.5)	453 (24.5)	220 (11.9)	1179 (63.7)	1.20 (1.05 1.37)	1.04 (0.89 1.21)
Incontinent	1486 (2.0)	282 (19.0)	155 (10.4)	1049 (70.6)	1.02 (0.87 1.19)	0.98 (0.78 1.24)
Ftigue^n^						
None	20,795 (27.6)	10,333 (49.7)	2008 (96.6)	8454 (40.7)	1 (Reference)	1 (Reference)
Minimal to Moderate	43,895 (58.2)	18,749 (42.7)	4587 (10.4)	20,559 (46.8)	1.10 (1.04 1.16)	1.03 (0.97 1.09)
Severe	10,790 (14.3)	2673 (24.8)	819 (7.6)	7298 (67.6)	0.77 (0.71 0.84)	0.81 (0.74 0.89)
Difficult or unable to move self to standing^m^						
Not present	46,643 (61.8)	21,862 (46.9)	4196 (9.0)	20,585 (44.1)	1 (Reference)	1 (Reference)
Present	28,838 (38.2)	9894 (34.3)	3218 (11.2)	15,726 (54.5)	1.22 (1.17 1.28)	1.03 (0.97 1.09)
Dizziness^m^						
Not present	63,907 (84.7)	27,112 (42.4)	6315 (9.9)	30,480 (47.7)	1 (Reference)	1 (Reference)
Present	11,574 (15.3)	4644 (40.1)	1099 (9.5)	5831 (50.4)	0.95 (0.89 1.01)	0.97 (0.91 1.04)
Unsteady Gait^m^						
Not present	35,820 (47.5)	16,750 (46.8)	3076 (8.6)	15,994 (44.7)	1 (Reference)	1 (Reference)
Present	39,661 (52.5)	15,006 (37.8)	4338 (10.9)	20,317 (51.2)	1.27 (1.21 1.32)	1.07 (1.01 1.13)
Previous Fall^m^						
No Fall	44,353 (58.8)	20,101 (45.3)	3484 (7.9)	20,768 (46.8)	1 (Reference)	1 (Reference)
Had at least one fall	31,128 (41.2)	11,655 (37.4)	3930 (12.6)	15,543 (49.9)	1.65 (1.58 1.73)	1.24 (1.18 1.30)
Easily Distracted^c^						
Not present	57,382 (76.0)	24,359 (42.1)	5618 (9.8)	27,405 (47.8)	1 (Reference)	1 (Reference)
Present	18,080 (24.0)	7398 (40.9)	1795 (9.9)	8887 (49.2)	1.05 (0.99 1.11)	1.04 (0.87 1.11)
Mental Function Varies over the course of a day^c^						
Not present	58,323 (77.3)	25,326 (43.4)	5688 (9.8)	27,309 (46.8)	1 (Reference)	1 (Reference)
Present	17,139 (22.7)	6431 (37.5)	1725 (10.1)	8983 (52.4)	1.04 (0.99 1.10)	0.99 (0.93 1.06)
Wandering^f^						
Not Present	72,470 (96.0)	30,658 (42.3)	7133 (9.8)	34,679 (47.9)	1 (Reference)	1 (Reference)
Present	2993 (4.0)	1099 (36.7)	280 (9.4)	1614 (53.9)	0.94 (0.83 1.06)	0.97 (0.85 1.10)
Frequency of Pain^n^						
No pain	30,029 (39.8)	12,096 (40.3)	2705 (9.0)	15,228 (50.7)	1 (Reference)	1 (Reference)
Not in last 3 days	7944 (10.5)	3448 (43.4)	761 (9.6)	3735 (47.0)	1.10 (1.02 1.19)	1.03 (0.89 1.19)
At least once in last 3 days	37,507 (49.7)	16,211 (43.2)	3948 (10.5)	17,348 (46.3)	1.20 (1.14 1.26)	1.07 (0.91 1.24)
Intensity of Highest level of Pain^n^						
None	30,640 (40.6)	12,382 (40.4)	2750 (9.0)	15,508 (50.6)	1 (Reference)	1 (Reference)
Mild to Moderate	33,598 (44.5)	14,483 (43.1)	3569 (10.6)	15,546 (46.3)	1.21 (1.15 1.27)	0.99 (0.83 1.17)
Severe to Excruciating	11,242 (14.9)	4890 (43.5)	1095 (9.7)	5257 (46.8)	1.09 (1.02 1.17)	0.97 (0.81 1.17)
Consistency of Pain^n^						
None/Very Little	32,872 (43.6)	13,324 (40.5)	2960 (9.0)	16,588 (50.5)	1 (Reference)	1 (Reference)
Intermittent	33,257 (44.1)	14,281 (42.9)	3534 (10.6)	15,442 (46.4)	1.22 (1.16 1.28)	1.04 (0.92 1.17)
Constant	9351 (12.4)	4150 (44.4)	920 (9.8)	4281 (45.8)	1.07 (1.00 1.15)	1.00 (0.87 1.15)
Body Mass Index						
Underweight	4287 (5.7)	1286 (30.0)	584 (13.6)	2417 (56.4)	1 (Reference)	1.20 (1.09 1.32)
Normal	27,185 (36.0)	11,461 (41.2)	2762 (10.2)	12,962 (47.7)	1.34 (1.23 1.47)	1 (Reference)
Overweight	9828 (13.0)	5042 (51.3)	747 (7.6)	4039 (41.1)	0.75 (0.69 0.81)	0.82 (0.75 0.89)
Obese	7224 (9.6)	4136 (57.3)	450 (6.3)	2638 (36.5)	0.63 (0.57 0.69)	0.74 (0.67 0.82)
Unknown	26,960 (35.7)	9833 (36.5)	2871 (10.7)	14,256 (52.9)	0.96 (0.91 1.01)	0.95 (0.90 1.00)
Smokes tobacco daily^n^						
No	71,319 (94.5)	30,017 (42.1)	7034 (9.9)	34,268 (48.0)	1 (Reference)	1 (Reference)
Yes	4161 (5.5)	1738 (41.8)	380 (9.1)	2043 (49.1)	0.94 (0.85 1.04)	1.18 (1.06 1.31)
Consumes Alcohol^n^						
None	60,295 (79.9)	24,669 (40.9)	6049 (10.0)	29,577 (49.1)	1 (Reference)	1 (Reference)
At least one drink	15,185 (20.1)	7086 (46.7)	1365 (9.0)	6734 (44.3)	0.91 (0.86 0.96)	1.03 (0.96 1.09)
Weight Loss of 5% or more^n^						
No	62,811 (83.2)	27,949 (44.5)	6191 (9.9)	28,671 (45.6)	1 (Reference)	1 (Reference)
Yes	12,669 (16.8)	3806 (30.0)	1223 (9.7)	7640 (60.3)	0.98 (0.92 1.04)	0.98 (0.91 1.05)
Dehydrated^n^						
No	73,883 (97.9)	31,454 (42.6)	7232 (9.8)	35,197 (47.6)	1 (Reference)	1 (Reference)
Yes	1597 (2.1)	301 (18.8)	182 (11.4)	1114 (69.8)	1.10 (0.95 1.27)	0.92 (0.78 1.08)
Decrease in food/fluid consumed^n^						
No	65,999 (87.4)	29,110 (44.1)	6549 (9.9)	30,340 (46.0)	1 (Reference)	1 (Reference)
Yes	9481 (12.6)	2645 (27.9)	865 (9.1)	5971 (63.0)	0.92 (0.86 0.99)	0.89 (0.82 0.96)
Parkinson’s Disease^m^						
Not present	72,706 (96.3)	30,638 (42.1)	7084 (9.7)	34,984 (48.1)	1 (Reference)	1 (Reference)
Diagnosis present	2775 (3.7)	1118 (40.3)	330 (11.9)	1327 (47.8)	1.21 (1.08 1.35)	1.18 (1.05 1.32)
Stroke/CVA^m^						
Not Present	62,801 (83.2)	26,766 (42.6)	5991 (9.5)	30,044 (47.8)	1 (Reference)	1 (Reference)
Diagnosis present	12,680 (16.8)	4990 (39.4)	1423 (11.2)	6267 (49.4)	1.15 (1.09 1.22)	1.13 (1.06 1.20)
COPD^m^						
Not present	63,678 (84.4)	27,856 (43.7)	6414 (10.1)	29,408 (46.2)	1 (Reference)	1 (Reference)
Diagnosis present	11,803 (15.6)	3900 (33.0)	1000 (8.5)	6903 (58.5)	0.81 (0.76 0.86)	1.02 (0.95 1.10)
Cancer^m^						
Not Present	63,455 (84.1)	28,612 (45.1)	6576 (10.4)	28,267 (44.5)	1 (Reference)	1 (Reference)
Diagnosis present	12,026 (15.9)	3144 (26.1)	838 (7.0)	8044 (66.9)	0.65 (0.61 0.70)	0.73 (0.67 0.78)
Dyspnoea^n^						
Not present	39,296 (52.1)	18,321 (46.6)	4280 (10.9)	16,695 (42.5)	1 (Reference)	1 (Reference)
Absent at rest	30,415 (40.3)	12,123 (39.9)	2781 (9.1)	15,511 (51.0)	0.83 (0.79 0.87)	0.86 (0.82 0.91)
Present at rest	5769 (7.6)	1311 (22.7)	353 (6.1)	4105 (71.2)	0.53 (0.47 0.59)	0.62 (0.55 0.71)
Environment^o^						
No	67,671 (89.7)	28,612 (42.3)	6617 (9.8)	32,442 (47.9)	1 (Reference)	1 (Reference)
Yes	7804 (10.3)	3141 (40.2)	797 (10.2)	3866 (49.5)	1.09 (1.00 1.17)	1.05 (0.97 1.14)

^b^1 value missing, ^c^22 values missing, ^d^18 values missing, ^e^19 values missing, ^f^21 values missing, ^g^3008 values missing, ^h^1596 values missing, ^i^2 values missing, ^j^61 values missing, ^k^215 values missing, ^m^3 values missing, ^n^4 values missing, ^o^9 values missing

*Adjusted for age, sex and ethnicity

### Injuries excluding hip fracture

Table 4 presents the adjusted SHRs after removing hip fractures from the analyses. After removing hip fractures from the injuries sustained, female sex, age, previous falls, tobacco use, and being underweight were associated with an increased risk of injury. High BMI and dyspnoea were associated with a reduced risk of injury. Females were 1.3 times more likely to sustain an injury than males (SHR 1.30 95% CI: 1.19–1.42). Compared to people who had no dyspnoea, individuals who had dyspnoea at rest were the least likely to sustain an injury (SHR: 0.54 95% CI: 0.44–0.67).

**Table 4:** Adjusted competing risk regression with hip fractures censored from analysis

Variable names	Adjusted SHR (95% Confidence Interval)
Sex	
Male	1 (Reference)
Female	1.30 (1.19 1.42)
Age Group (years)	
65–74	1 (Reference)
75–84	1.34 (1.17 1.54)
85–94	1.55 (1.34 1.79)
95+	1.44 (1.10 1.89)
Ethnicity	
Māori	1 (Reference)
Pacific People	0.69 (0.47 1.01)
Asian	1.03 (0.73 1.45)
European	1.32 (1.06 1.63)
Other	1.40 (0.89 2.20)
Living Arrangement	
Lives alone	1 (Reference)
Lives with others	0.93 (0.85 1.01)
Cognitive Skills	
Independent	1 (Reference)
Minimal Independence	0.98 (0.90 1.08)
Moderate to Severe dependence	0.93 (0.80107)
No discernible consciousness	0.02 (0.01 0.04)
Hearing loss	
Adequate	1 (Reference)
Minimal to moderate	1.02 (0.94 1.11)
Severe to none	0.96 (0.79 1.18)
Vision loss	
Adequate	1 (Reference)
Minimal to moderate	0.97 (0.88 1.06)
Severe to none	1.10 (0.87 1.39)
Walking	
Independent	1 (Reference)
Some assistance required	1.06 (0.85 1.32)
Maximum Assistance/Dependent	1.57 (1.12 2.20)
Locomotion	
Independent	1 (Reference)
Some assistance required	1.19 (0.95 1.48)
Dependent	0.83 (0.60 1.17)
Primary Mode of Locomotion	
Walking, no assistive device	1 (Reference)
Assisted walking	1.20 (1.08 1.33)
Unable to walk	0.90 (0.62 1.30)
Timed 4 Metre walk	
0–15 s	1 (Reference)
16–29 s	1.21 (1.06 1.38)
30+ seconds	1.10 (0.95 1.27)
Incomplete test	1.08 (0.96 1.21)
Total hours of exercise or physical activity	
None/Less than 1 h	1 (Reference)
1–4 h	1.06 (0.98 1.16)
4 h or more	1.05 (0.86 1.28)
Number of days left house in last 3 days	
None	1 (Reference)
1–2 days	0.95 (0.85 1.05)
3 days	0.88 (0.79 0.99)
Bladder Continence	
Continent	1 (Reference)
Infrequently incontinent	1.01 (0.88 1.15)
Occasionally incontinent	1.08 (0.95 1.24)
Frequently Incontinent	1.05 (0.93 1.18)
Incontinent	1.21 (0.87 1.45)
Bowel Continence	
Continent	1 (Reference)
Infrequently incontinent	0.99 (0.84 1.16)
Occasionally Incontinent	0.92 (0.77 1.11)
Frequently Incontinent	1.07 (0.83 1.39)
Incontinent	1.09 (0.73 1.61)
Fatigue	
None	1 (Reference)
Minimal to Moderate	1.04 (0.94 1.14)
Severe	0.78 (0.66 0.92)
Difficult or unable to move self to standing	
Not present	1 (Reference)
Present	0.95 (0.86 1.05)
Dizziness	
Not present	1 (Reference)
Present	0.97 (0.86 1.08)
Unsteady Gait	
Not present	1 (Reference)
Present	1.05 (0.96 1.15)
Previous Fall	
No Fall	1 (Reference)
Had at least one fall	1.39 (1.29 1.51)
Easily Distracted	
Not present	1 (Reference)
Present	1.09 (0.98 1.21)
Mental Function Varies over the course of a day	
Not present	1 (Reference)
Present	0.99 (0.88 1.10)
Wandering	
Not Present	1 (Reference)
Present	0.87 (0.70 1.08)
Frequency of Pain	
No pain	1 (Reference)
Not in last 3 days	1.15 (0.90 1.48)
At least once in last 3 days	1.25 (0.95 1.63)
Intensity of Highest level of Pain	
None	1 (Reference)
Mild to Moderate	0.96 (0.72 1.26)
Severe to Excruciating	0.88 (0.65 1.19)
Consistency of Pain	
None/Very Little	1 (Reference)
Intermittent	1.01 (0.82 1.24)
Constant	1.01 (0.80 1.28)
Body Mass Index	
Underweight	1.35 (1.15 1.57)
Normal	1 (Reference)
Overweight	0.79 (0.69 0.91)
Obese	0.75 (0.63 0.89)
Unknown	0.93 (0.85 1.02)
Smokes tobacco daily	
No	1 (Reference)
Yes	1.24 (1.04 1.49)
Consumes Alcohol	
None	1 (Reference)
At least one drink	1.01 (0.91 1.23)
Weight Loss of 5% or more	
No	1 (Reference)
Yes	0.99 (0.88 1.11)
Dehydrated	
No	1 (Reference)
Yes	1.15 (0.89 1.48)
Decrease in food/fluid consumed	
No	1 (Reference)
Yes	0.88 (0.77 1.01)
Parkinson’s Disease	
Not present	1 (Reference)
Diagnosis present	0.97 (0.85 1.27)
Stroke/CVA	
Not Present	1 (Reference)
Diagnosis present	1.08 (0.97 1.97)
COPD	
Not present	1 (Reference)
Diagnosis present	1.04 (0.92 1.18)
Cancer	
Not Present	1 (Reference)
Diagnosis present	0.72 (0.63 0.82)
Dyspnoea	
Not present	1 (Reference)
Absent at rest	0.82 (0.75 0.89)
Present at rest	0.54 (0.44 0.67)
Environment	
No	1 (Reference)
Yes	1.00 (0.87 1.15)

## Discussion

### Key findings

There were a total of 7414 (9.8%) people who sustained a falls-related injury. We identified female sex, older age, living alone, a diagnosis of Parkinson’s disease, stroke/CVA, previous falls, unsteady gait, tobacco use, and being underweight were associated with an increased risk of sustaining an injury. In contrast, a reduced risk of injury was associated with a diagnosis of cancer, dyspnoea, high BMI, and a decrease in the amount of food or fluid usually consumed. Two mobility-related risk factors had a non-monotonic association with injury (Primary Mode of Locomotion and Timed 4 Metre walk) both suggesting that those unable to walk were not significantly associated with fall-related injury.

Hip fractures comprised nearly two-thirds (63.9%) of all injuries sustained by older people, and when these were removed from analysis there were fewer factors associated with injury. The factors that were associated after hip fractures were censored were sex, age, previous falls, dyspnoea, tobacco use, and BMI.

### Findings within the literature

Co-morbidities were associated with fall-related injuries. Stroke/CVA and Parkinson’s disease were both associated with an increased risk of injury. Previous studies have shown that stroke/CVA is associated with an increase in falls risk [38, 39]. Additionally, several studies have identified that individual’s with Parkinson’s disease are at an elevated risk of falling and sustaining an injury [40]. Our study found that cancer was associated with a reduced risk of injury. However, many studies exploring the risk of falls and fall-related injuries in cancer patients have identified that individual’s with cancer were at an elevated risk of falling and sustaining an injury [41, 42]. Schluter et al. included cancer in an adjusted model for identifying if incontinence was associated with falls within an interRAI-HC cohort. Their model showed that people with cancer had a reduced risk of falls [43]. In an interRAI-HC setting individuals with cancer appear to be at a reduced risk of falls and fall-related injuries. This is possibly due to behavioural differences with cancer patients being less likely to take part in activities such as walking that can lead to falls and falls-related injuries. This could also explain the lower risk in individuals who experience dyspnoea, as they are less likely to undertake extraneous activities and therefore be at a reduced risk of falling.

BMI was associated with fall-related injuries. Those who were underweight were 1.2 times more likely to sustain a fall-related injury than people with a normal BMI. Previous research has suggested that those with a low BMI are more likely to be at risk of multiple types of fractures [44]. It has also been noted in the literature that those who are overweight and obese are at a reduced risk of sustaining an injury [44, 45].

Environmental factors such as disrepair of the home, and limited access to rooms were not associated with fall-related injuries in the interRAI-HC. This may be due to the limited number of people who have such problems with their home environment. Previous studies have identified items common to homes such as hard-flooring, loose rugs, and inadequate lighting as risk factors for falls and fall-related injuries [46, 47].

### Limitations of the study

Osteoporosis is a well-known risk factor for many bone fractures and this was unable to be included as a variable. The New Zealand interRAI-HC version does not contain osteoporosis as a diagnosis; therefore, we were unable to identify anyone who had a diagnosis of osteoporosis within the cohort. Additionally, the BMI data for many individuals were missing with approximately 35.7% of individuals having no recorded BMI. This is most likely because it is difficult to measure height and weight data for frail individuals, particularly as many people in this cohort have mobility issues and stepping up onto a scale may be challenging. The results show BMI, was significantly associated with injuries suggesting that BMI is an important risk factor and may have clinical implications.

Ecodes were unavailable for a number of hospital admissions information. This means that some of the injuries sustained may not have occurred due to a fall. However, the injuries chosen are common falls-related injuries, so we anticipate that most injuries included were sustained from a fall.

Our study used New Zealand data of older people who require health care services and may not be generalizable to a larger international audience or to a healthy population of New Zealand older adults. However, the results may be generalizable to other home care cohorts internationally.

### Summary of the implications of the work for practice and research

The New Zealand MoH has implemented the New Zealand healthy ageing strategy to provide support for people with high and complex needs, including those who require home care services, so they can live as independently as possible [48, 49]. The interRAI-HC is a useful tool for determining the appropriate care programme for each individual. Fall-related injuries lead to reduced quality of life and significant disability for older adults. While falls prevention programmes are being implemented, it is also important to reduce the risk of serious injury after a fall [23]. Determining risk factors for a range of fall-related injuries can allow for targeted interventions to help reduce injuries sustained. Additional work will also be conducted to develop an injury risk score to identify those who may be at an elevated risk of sustaining a falls-related injury. While there are already well established injury prediction models such as the FRAX and the Garvan, an injury score developed for a frail community-dwelling cohort will more precisely predict who is at risk of injury [50–52]. Additionally, the FRAX and Garvan scores predict the 5- and 10-year risk of hip fracture, whereas the home care cohort may be unlikely to live that long, so a 1- or 2-year risk of injury would be better utilised for this population.

## Conclusions

Risk factors for injuries were female sex, older age, living alone, Parkinson’s disease, stroke/CVA, falls, unsteady gait, tobacco use, and being underweight. Cancer, dyspnoea, high BMI, and a decrease in the amount of food or fluid usually consumed, were associated with a reduced risk of sustaining an injury. While it is important to reduce the risk of falls, it is also important to reduce the risk of falls-related injuries. Knowing risk factors associated with these types of injuries can help to develop focused intervention programmes. Additionally, development of a predictive model to identify those who would benefit from intervention programmes would be beneficial.

## Data Availability

The data that support the findings of this study are available from Technical Advisory Services (TAS) but restrictions apply to the availability of these data, which were used under license for the current study, and so are not publicly available. Anyone wishing to access the data must apply to TAS following the guidelines provided on their website https://www.interrai.co.nz/data-research-and-reporting/requesting-interrai-data/.

## Abbreviations

ARC: Aged Residential Care
BMD: Bone Mineral Density
BMI: Body Mass Index
CI: Confidence Interval
COPD: Chronic Obstructive Pulmonary Disease
CPS: Cognitive Performance Scale
CVA: Cerebrovascular Accident
DBI: Drug Burden Index
ICD-10 AM: International Statistical Classification of Diseases and Related Health Problems, Tenth Revision, Australian Modification
interRAI-HC: international Residential Assessment Instrument, Home Care
MDS: Minimum Dataset
MoH: Ministry of Health
MORT: Mortality Collection
NHI: National Health Index
NMDS: National Minimum Dataset
RECORD: REporting of Studies Conducted using Observational Routinely collected Data
SHR: Subhazard Ratio
TAS: Technical Advisory Services

## References

[1] Kannus P, Sievänen H, Palvanen M, Järvinen T, Parkkari J (2005). Prevention of falls and consequent injuries in elderly people. Lancet..

[2] Keller JM, Sciadini MF, Sinclair E, O'Toole RV (2012). Geriatric trauma: demographics, injuries, and mortality. J Orthop Trauma.

[3] Hildebrand F, Pape HC, Horst K, Andruszkow H, Kobbe P, Simon TP, Marx G, Schürholz T (2016). Impact of age on the clinical outcomes of major trauma. Eur J Trauma Emerg Surg.

[4] Kannus P, Parkkari J, Koskinen S, Niemi S, Palvanen M, Järvinen M, Vuori I (1999). Fall-induced injuries and deaths among older adults. JAMA..

[5] Hill K, Kerse N, Lentini F, Gilsenan B, Osborne D, Browning C, Harrison J, Andrews G (2002). Falls: a comparison of trends in community, hospital and mortality data in older Australians. Aging Clin Exp Res.

[6] Lord S, Sherrington C, Menz H, Close J (2007). Epidemiology of falls and fall-related injuries. Falls in older people: risk factors and strategies for prevention.

[7] English, B. Speech to the Treasury guest lecture series on social investment. 2016. https://www.beehive.govt.nz/speech/speech-treasury-guest-lecture-series-social-investment. Accessed June 2020.

[8] Morris JN, interRAI: InterRAI Home Care (HC) Assessment Form and User's Manual: interRAI; 2010.

[9] Ryall T. Care assessments improving rest home care. 2013. Available from http://www.beehive.govt.nz/release/care-assessments-improving-rest-home-care. Accessed 5 July 2020.

[10] Nevitt MC, Cummings SR, Kidd S, Black D (1989). Risk factors for recurrent nonsyncopal falls: a prospective study. JAMA..

[11] Close J, Ellis M, Hooper R, Glucksman E, Jackson S, Swift C (1999). Prevention of falls in the elderly trial (PROFET): a randomised controlled trial. Lancet..

[12] Davies AJ, Kenny RA (1996). Falls presenting to the accident and emergency department: types of presentation and risk factor profile. Age Ageing.

[13] Mahoney J, Sager M, Dunham NC, Johnson J (1994). Risk of falls after hospital discharge. J Am Geriatr Soc.

[14] Jørgensen L, Engstad T, Jacobsen BK (2002). Higher incidence of falls in long-term stroke survivors than in population controls: depressive symptoms predict falls after stroke. Stroke..

[15] Tinetti ME, Speechley M, Ginter SF (1988). Risk factors for falls among elderly persons living in the community. N Engl J Med.

[16] van Dijk PT, Meulenberg OG, Van de Sande HJ, Habbema JD (1993). Falls in dementia patients. Gerontologist..

[17] Sturnieks DL, Tiedemann A, Chapman K, Munro B, Murray SM, Lord SR (2004). Physiological risk factors for falls in older people with lower limb arthritis. J Rheumatol.

[18] Schwartz AV, Hillier TA, Sellmeyer DE, Resnick HE, Gregg E, Ensrud KE, Schreiner PJ, Margolis KL, Cauley JA, Nevitt MC, Black DM (2002). Older women with diabetes have a higher risk of falls: a prospective study. Diabetes Care.

[19] Barber J, Mills H, Horne G, Purdie G, Devane P (1995). The incidence of hip fractures in Maori and non-Maori in New Zealand. N Z Med J.

[20] Schluter PJ, Arnold EP, Jamieson HA. Falls and hip fractures associated with urinary incontinence among older men and women with complex needs: a national population study. Neurourol Urodyn. 2018; 37(4):1336–43. doi: 10.1002/nau.23442 10.1002/nau.23442 29130513

[21] Jamieson HA, Nishtala PS, Scrase R, Deely JM, Abey-Nesbit R, Hilmer SN, Abernethy DR, Berry SD, Mor V, Lacey CJ, Schluter PJ (2019). Drug burden index and its association with hip fracture among older adults: a national population-based study. J Gerontol A Med Sci.

[22] Nishtala PS, Chyou TY (2017). Zopiclone use and risk of fractures in older people: population-based study. JAMDA..

[23] Hanger HC (2017). Low-impact flooring: does it reduce fall-related injuries?. JAMDA..

[24] Elley CR, Robertson MC, Garrett S, Kerse NM, McKinlay E, Lawton B, Moriarty H, Moyes SA, Campbell AJ (2008). Effectiveness of a falls-and-fracture nurse coordinator to reduce falls: a randomized, controlled trial of at-risk older adults. J Am Geriatr Soc.

[25] Campbell AJ, Robertson MC, Gardner MM, Norton RN, Tilyard MW, Buchner DM (1997). Randomised controlled trial of a general practice programme of home based exercise to prevent falls in elderly women. BMJ..

[26] Health Quality & Safety Commission New Zealand. Reducing harm from falls: recommended evidence-based resources. Wellington: HQSC;2019. p. 7–11. Available from: https://www.hqsc.govt.nz/assets/Falls/PR/falls-2019-evidence-base-final.pdf. Accessed 30 Aug 2020.

[27] Schluter PJ, Ahuriri-Driscoll A, Anderson TJ, Beere P, Brown J, Dalrymple-Alford J, David T, Davidson A, Gillon DA, Hirdes J, Keeling S (2016). Comprehensive clinical assessment of home-based older persons within New Zealand: an epidemiological profile of a national cross-section. Aust NZ J Publ Heal.

[28] Hirdes JP, Ljunggren G, Morris JN, Frijters DH, Soveri HF, Gray L, Björkgren M, Gilgen R (2008). Reliability of the interRAI suite of assessment instruments: a 12-country study of an integrated health information system. BMC Health Serv Res.

[29] Ministry of Health*.* Comprehensive Clinical Assessment for Aged Care (interRAI) . 2014. Available from: http://ithealthboard.health.nz/our-programmes/common-clinical-information/comprehensive-clinical-assessment-aged-care-interrai. Accessed 30 Aug 2020.

[30] Ministry of Health. National Health Index Overview. 2009. Available from: https://www.health.govt.nz/our-work/health-identity/national-health-index/national-health-index-overview. Accessed 5 July 2020.

[31] Poutasi K. Ethnicity data protocols for the health and disability sector. Wellington: Ministry of Health. 2004. Available from: https://www.health.govt.nz/publication/hiso-100012017-ethnicity-data-protocols. Accessed 5 July 2020.

[32] Ministry of Health. National Minimum Dataset (hospital events). 2019. Available from: https://www.health.govt.nz/nz-health-statistics/national-collections-and-surveys/collections/national-minimum-dataset-hospital-events. Accessed 5 July 2020.

[33] Ministry of Health. Mortality Collection. 2019. Available from: https://www.health.govt.nz/nz-health-statistics/national-collections-and-surveys/collections/mortality-collection. Accessed 5 July 2020.

[34] Benchimol EI, Smeeth L, Guttmann A, Harron K, Moher D, Petersen I, Sørensen HT, von Elm E, Langan SM, RECORD Working Committee. The REporting of studies Conducted using Observational Routinely-collected health Data (RECORD) statement. PLoS Med. 2015; 12(10):e1001885. doi: 10.1371/journal.pmed.1001885 10.1371/journal.pmed.1001885 PMC4595218 26440803

[35] Fine JP, Gray RJ (1999). A proportional hazards model for the subdistribution of a competing risk. J Am Stat Assoc.

[36] IBM Corp. Released 2017. IBM SPSS statistics for windows, version 25.0. Armonk: IBM Corp.

[37] StataCorp (2019). Stata statistical software: release 16.

[38] Batchelor FA, Mackintosh SF, Said CM, Hill KD (2012). Falls after stroke. Int J Stroke.

[39] Tan KM, Tan MP (2016). Stroke and falls—clash of the two titans in geriatrics. Geriatrics..

[40] Stevens JA (2005). Falls among older adults—risk factors and prevention strategies. J Saf Res.

[41] Wildes TM, Dua P, Fowler SA, Miller JP, Carpenter CR, Avidan MS, Stark S (2015). Systematic review of falls in older adults with cancer. J Geriatr Oncol.

[42] Fasano A, Canning CG, Hausdorff JM, Lord S, Rochester L (2017). Falls in Parkinson's disease: a complex and evolving picture. Mov Disord.

[43] Schluter PJ, Askew DA, Jamieson HA, Arnold EP (2020). Urinary and fecal incontinence are independently associated with falls risk among older women and men with complex needs: a national population study. Neurourol Urodynam.

[44] De Laet C, Kanis JA, Odén A, Johanson H, Johnell O, Delmas P, Eisman JA, Kroger H, Fujiwara S, Garnero P, McCloskey EV (2005). Body mass index as a predictor of fracture risk: a meta-analysis. Osteoporosis Int.

[45] Galvard H, Elmståhl S, Elmståhl B, Samuelsson SM, Robertsson E (1996). Differences in body composition between female geriatric hip fracture patients and healthy controls: body fat is more important as explanatory factor for the fracture than body weight and lean body mass. Aging Clin Exp Res.

[46] Lord SR, Menz HB, Sherrington C. Home environment risk factors for falls in older people and the efficacy of home modifications. Age Ageing. 2006; 35(suppl_2):55–9. doi: 10.1093/ageing/afl088 10.1093/ageing/afl088 16926207

[47] Cesari M, Landi F, Torre S, Onder G, Lattanzio F, Bernabei R (2002). Prevalence and risk factors for falls in an older community-dwelling population. J Gerontol A Med Sci..

[48] Ministry of Health. New Zealand Health Strategy: Future Direction. 2016. p. 1–36. Available from: https://www.health.govt.nz/system/files/documents/publications/new-zealand-health-strategy-futuredirection-2016-apr16.pdf. Accessed 5 July 2020.

[49] Ministry of Health. New Zealand Health Strategy: Roadmap of actions 2016. 2016. p. 4–25. Available from: https://www.health.govt.nz/new-zealand-health-system/new-zealand-health-strategy-roadmap-actions-2016. Accessed 5 July 2020.

[50] Centre for Metabolic Bone Diseases. University of Sheffield. Sheffield: University of Sheffield; 2008. Frax Fracture risk assessment tool. Available from: https://www.sheffield.ac.uk/FRAX/tool.aspx?country=9. Accessed 30 Aug 2020.

[51] Nguyen ND, Frost S, Center JR, Eisman JA, Nguyen T (2007). Development of a nomogram for individualizing hip fracture risk in men and women. Osteoporosis Int..

[52] Nguyen ND, Frost SA, Center J, Eisman JA, Nguyen TV (2008). Development of prognostic nomograms for individualizing 5-year and 10-year fracture risks. Osteoporosis Int.

